# Fourth Branchial Cleft Cyst: An Important Differential Diagnosis in Lateral Neck Masses

**DOI:** 10.7759/cureus.36913

**Published:** 2023-03-30

**Authors:** Miguel A Aristizabal

**Affiliations:** 1 Aesthetic Medicine, ADEI - Aesthetics & Dermatology Institute, Bogota, COL

**Keywords:** branchial apparatus, branchial cyst, branchial cleft anomaly, branchial, branchial cleft cyst

## Abstract

Branchial anomalies are a result of abnormal development during embryogenesis and are a rare cause of lateral neck masses of congenital origin. The second branchial cleft is the most common site of origin, with abnormalities arising from the first, third, and fourth clefts being rarer. Although cysts originating from branchial clefts are infrequent, it is important to consider this pathology in the differential diagnosis of neck masses, particularly those located laterally. This article discusses a rare case of a 49-year-old female patient who presented with the sudden appearance of a lateral neck mass after sports practice. The patient underwent extensive diagnostic studies, including radiological studies, which were compatible with a fourth branchial cleft cyst. The patient remains asymptomatic, and possible surgical treatment is being evaluated by the head and neck surgery service. This clinical case highlights the importance of prompt diagnosis and appropriate management of rare pathologies such as branchial cleft cysts.

## Introduction

The branchial arches or gill apparatus contribute to the proper formation of the head and neck during the fourth week of gestation, these arches are composed of ectodermal clefts and endodermal pouches. Congenital anomalies of the ectodermal clefts of the branchial arches occur due to incomplete obliteration, resulting in a cyst in the majority of cases (75%) or a sinus (25%) [1].

These alterations do not have a predilection for sex and can occur at any age, although they are more common in adolescents and young adults [2]. Usually, these cysts are unilateral and the arch typically affected is the second branchial arch in 90-95% of cases, with only 2% corresponding to anomalies of the fourth branchial arch [3].

Here I report the case of a young adult patient with a neck mass with unusual characteristics and locations, later identified as a fourth branchial cleft anomaly, an exceptional and infrequent case.

## Case presentation

This is a 49-year-old female patient with no relevant medical history, originally from Holland and currently residing in the city of Bogotá. She suddenly presented with pain and a sensation of a mass in the right posterior neck region after engaging in physical activity. One month after this episode, the symptoms remained unchanged, and she denied experiencing any other symptoms. It is important to consider that she has not had similar episodes in the past.

**Figure 1:**
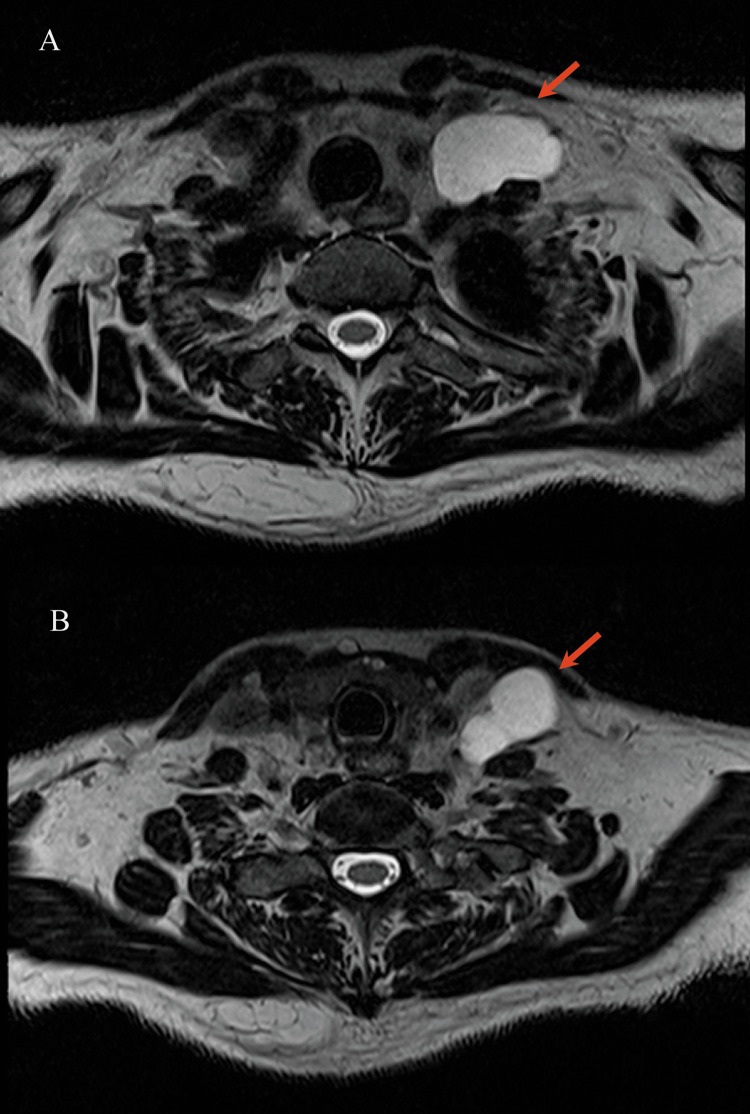
Contrast-enhanced MRI showing the cyst located on the right side behind the jugular vein and lateral to the thyroid gland (arrow). MRI: magnetic resonance imaging

Upon physical examination, an overweight female patient was found to have a soft, mobile, painless mass on the right side of her neck with no additional masses or lymphadenopathy present. The rest of the physical exam was within normal limits. The patient underwent a contrast-enhanced neck magnetic resonance imaging (MRI) study, which revealed a large cyst possibly derived from the fourth branchial cleft on the right side, behind the jugular vein and lateral to the thyroid, almost submerged in that area, with smooth and thin walls (Figure 1).

Following diagnosis, the patient received information about the cause, course, and prognosis of the disease and was referred to the head and neck surgery department to explore possible surgical options.

## Discussion

Different specific structures of the neck are derived from the branchial arches. Those derived from the fourth branchial arch correspond to the superior parathyroid glands, laryngeal cartilages, pharyngeal and laryngeal constrictor muscles, the superior laryngeal nerve, the left thoracic aorta, the proximal right subclavian artery, and the last branchial body that forms the parafollicular cells of the thyroid. Failure of obliteration of the pharyngobranchial duct and the fourth branchial cleft results in an alteration of neck anatomy [4].

Branchial cleft anomalies comprise 30% of congenital neck masses, and only 2% are fourth branchial cleft anomalies, which are extremely rare. Fourth branchial cleft anomalies usually manifest as cysts in adults and as sinuses or fistulas in the pediatric population. These laryngotracheal sinus tracts begin at the apex of the piriform sinus and extend inferiorly to exit the pharynx through the lateral cricothyroid membrane. They then continue inferiorly to be located around the left aortic arch or right subclavian artery [5].

The clinical presentation of fourth branchial arch abnormalities varies with age. Episodes of dyspnea are the most common presentation in neonates [6], cervical cutaneous fistulas present in childhood, and later these cysts present classically with a repetitive history of infections and abscesses in the neck, usually on the left side in 93.6% of cases, on the right side in 6%, and bilaterally in 0.5%. Infectious episodes present clinically as persistent neck edema and may even involve the ipsilateral thyroid lobe to the location of the cyst, triggering acute suppurative thyroiditis. When these findings are present in the neck, a differential diagnosis should be made with subacute de Quervain's thyroiditis, Hashimoto's thyroiditis, or bleeding originating from a thyroid nodule [4].

In a review of 526 cases of congenital abnormalities of the fourth branchial arch, it was reported that 19% of cases are accompanied by bacterial superinfections as a result of the retrograde transmission of flora from the hypopharynx, where a variety of aerobic microorganisms have been found, mainly *Streptococcus*, *Staphylococcus*, *Haemophilus*, *Escherichia coli*, and anaerobic germs such as *Eikonella corrodens*, *Citrobacter*, and *Proteus *[7].

Although cysts or sinuses of the fourth branchial arch are a rare pathology, it is important to consider this anomaly as a differential diagnosis of lateral neck masses, along with others such as tumors of the carotid body, paragangliomas, dermoid cysts, thyroglossal duct cysts, neurofibromas, hemangiomas, lipomas, teratomas, and lymph node metastases [8].

A variety of diagnostic methods have been used to explore branchial cysts, among them, the most useful have been barium esophagogram and direct laryngoscopy, which have the best positive predictive value (between 88% and 90%, respectively). However, barium studies may not show a sinuous tract if there is inflammation, unlike laryngoscopy. Others such as MRI, computed tomography, and thyroid ultrasound have also been used to diagnose and evaluate cystic lesions. Many abnormalities are diagnosed as incidental findings [7].

Regarding treatment options, in the case of abscesses of the third and fourth branchial arches, incision and drainage are frequently performed, but have a high recurrence rate, up to 90%. The recommended treatment for cysts, sinuses, and fistulas is surgical excision along with partial thyroidectomy due to the risk of severe infection or primary carcinoma [9,10]. Surgery has a lower risk of recurrence, but a higher probability of complications or injury to cervical neurovascular structures, mainly in neonates and children [6].

Endoscopy with cauterization is a minimally invasive technique with fewer complications and costs. Other methods of cauterization have been described including monopolar electrocautery and chemocauterization with trichloroacetic acid [11].

In terms of complications arising from surgical and cauterization procedures, surgical site infections, salivary fistulas, and vocal cord paralysis have been reported in 5% to 6% of cases. During electrocautery, paralysis of the recurrent laryngeal nerve and the superior laryngeal nerve may occur due to inflammation and edema that can eventually compress these nerves. Although evidence is limited, most studies conclude that cauterization should be the primary treatment because it is minimally invasive, has a lower rate of complications, and can be performed simultaneously with other procedures such as incision and drainage in the case of an abscess [11,12]. Furthermore, successfully treated cases with sclerosing agents have been published [13].

## Conclusions

In conclusion, fourth branchial cleft anomalies are a rare but important differential diagnosis for lateral neck masses. Clinical presentations vary with age, and infectious episodes can lead to serious complications. Diagnosis is typically made using a combination of imaging and direct laryngoscopy, with surgical excision and partial thyroidectomy being the recommended treatment to prevent recurrence and reduce the risk of severe infection or primary carcinoma. Endoscopy with cauterization is a minimally invasive technique that may be preferred in certain cases due to its lower rate of complications. Overall, a thorough evaluation is necessary for any patient with unexplained neck masses, especially on the left side, to ensure prompt diagnosis and appropriate treatment.
